# Now is the time: the need to update guidance to expand access to *APOE* genetic testing

**DOI:** 10.3389/frdem.2026.1808512

**Published:** 2026-05-26

**Authors:** Wendy R. Uhlmann, Amanda K. Chan, Jill S. Goldman

**Affiliations:** 1 Division of Genetic Medicine, Department of Internal Medicine and Department of Human Genetics, University of Michigan, Ann Arbor, MI, United States; 2 Department of Neurology, Vagelos College of Physicians and Surgeons, Columbia University, New York, NY, United States

**Keywords:** Alzheimer’s disease, Alzheimer’s disease risk, *APOE* gene, genetic counseling (GC), genetic testing, *APOE* e4, dementia, clinical guidelines

## Abstract

Interest in *APOE* testing has increased due to an aging population, direct-to-consumer testing options, media coverage and FDA approval of anti-amyloid therapies. Previous Alzheimer’s disease (AD) guidelines cautioned against *APOE* testing in asymptomatic individuals. However, updated guidance is warranted due to the changing landscape and research demonstrating low adverse psychological outcomes of *APOE* testing. With testing for *APOE* now recommended prior to anti-amyloid therapies, more symptomatic individuals will be identified as having the higher risk ε4 variant. The children and siblings of the symptomatic individuals who test positive will have at least a 50% risk of also having an ε4 and may subsequently seek testing. Since testing for *APOE* is generally not clinically available to asymptomatic individuals, they may pursue direct-to-consumer genetic testing and, therefore, be tested without a clinician’s involvement or counseling. Given limitations and caveats of *APOE* testing, it is important that patients have access to education, counseling and testing in labs certified for clinical testing. Pre- and post-test education and counseling content for *APOE* testing, including risk figures, are provided in this Perspective. Both Alzheimer’s disease and cardiovascular disease risks associated with *APOE* should be addressed for all individuals undergoing testing. Given that it has been over a decade since *APOE* guidance was issued, the time has come for the medical community to discuss the changing landscape, expand *APOE* genetic testing access to individuals at higher risk, and update guidelines.

## Introduction

Fear of developing Alzheimer’s disease (AD) is a common aging concern, and genetic testing is frequently requested. More than 80 AD susceptibility genes have been identified with the Apolipoprotein E gene (*APOE*) being the first and conferring the strongest impact on risk for late-onset disease (>65 years old) (Serrano-Pozo et al., 2021). While the *APOE* ε4 variant is the most significant genetic AD risk factor, clinical guidelines have historically discouraged both diagnostic and predictive *APOE* testing because an ε4 variant is neither necessary nor sufficient for developing AD (American College of Medical Genetics/American Society of Human Genetics Working Group, 1995; Relkin et al., 1996; Waldemar et al., 2007; Goldman et al., 2011). However, the landscape around *APOE* testing is changing because: (1) there are demographic changes with a larger aging population at risk for AD (Alzheimer’s Association, 2025), (2) *APOE* testing is now recommended before utilizing anti-amyloid therapies (e.g., Lecanemab) given increased risk for ARIA (amyloid-related imaging abnormalities) associated with an ε4 variant (van Dyck et al., 2023), (3) *APOE* testing is recommended to assess cardiovascular disease (CVD) risk for individuals with possible dyslipidemia (Ison et al., 2025), (4) increased *APOE* testing of individuals who are symptomatic for AD and/or individuals with possible dyslipidemia will reveal the increased risk for being an *APOE* ε4 carrier to their asymptomatic children and siblings who may subsequently seek testing, (5) *APOE* genotype is frequently part of eligibility criteria for AD research and clinical trials (Langbaum et al., 2019), (6) there is a growing general population interest in learning AD risk due to media coverage (Breznican, 2022), and (7) testing can be found on the internet and accessed through direct-to-consumer (DTC) companies without clinician involvement. Guidelines for AD genetic testing were last issued in 2011 (Goldman et al., 2011). Given the changing landscape around *APOE* testing, this Perspective advocates for increasing access to clinical *APOE* testing, discusses key information to convey to patients, and recommends updating decades-old professional guidelines.

## Discussion

### *APOE*-associated risk

The *APOE* ε*4* allele is seen in approximately 60% of individuals with AD and 20–25% in the general population (Van Cauwenberghe et al., 2016). Based on longitudinal studies, the risk of clinical symptoms of mild cognitive impairment (MCI) and dementia by age 85 years is 10 to 15% without an *APOE* ε*4*, 20 to 25% with one ε4 allele, and 30 to 55% with two copies of ε4 though these estimates likely differ by ancestry (Qian et al., 2017; Belloy et al., 2023). Homozygosity for *APOE* ε4 is seen in approximately 2% of the general population and up to 15% of individuals with AD (Corder et al., 1993; Belloy et al., 2019). Of note, a 2024 study by Fortea et al. (2024) suggested that *APOE* ε4 homozygosity be considered a genetic form of AD, resulting in wide media attention and increased general population interest in *APOE* testing (Belluck, 2024; Neergaard, 2024). However, the Fortea et al. (2024) study was limited by assessment of only biological penetrance rather than clinical penetrance of symptoms and was not based on longitudinal studies that considered ethnicity and race (Reiman et al., 2025). *APOE* remains an AD susceptibility risk gene and is not deterministic unlike the highly penetrant *PSEN1, PSEN2* and *APP* genes that cause early-onset autosomal dominant AD.

### Clinical practice update needed for *APOE* testing

*APOE* genetic testing is not currently recommended for all symptomatic individuals since having an ε4 is neither necessary nor sufficient for AD. However, clinical *APOE* testing is recommended for symptomatic patients considering anti-amyloid therapies given increased ARIA risk if found to have an *APOE* ε4 variant (Cummings et al., 2022, 2023). An increase in *APOE* testing means that more symptomatic individuals will be identified as ε4 carriers. Children and siblings of ε4 carriers have at least a 50% risk of also having an ε4 (Table 1) and may subsequently seek testing. In addition, some individuals with actual or perceived cognitive symptoms may request *APOE* testing to have another data point about a possible AD diagnosis.

**Table 1:** Counseling recommendations for *APOE* genetic testing.

Counseling component	Content	Considerations and topics for discussion
Background information on *APOE* alleles and AD risk	General population risk for AD: 10–12% (Goldman et al., 2011) Risk for AD if first-degree relative affected: 20–25% (Bird, 2008) APOE alleles and AD association: ε2: protective ε3: neutral ε4: increased AD risk in dose-dependent manner Prevalence and risk of one *APOE* ε4: 60% of individuals with AD, 20–25% of individuals in general population 2-3x increased AD risk, similar risk to having a parent with AD (20–25% lifetime risk (Qian et al., 2017) Prevalence and risk of two *APOE* ε4: 15% of individuals with AD, 2% of individuals in general population Up to 15x increased AD risk (30–55% lifetime risk (Qian et al., 2017)	AD is a multifactorial (complex) condition, meaning both genetic and environmental factors are involved. *APOE* ε4 is just one of many genetic risk factors *APOE* risk may differ by ancestry (Belloy et al., 2023) *APOE* predictive value studies are limited and focus on the symptomatic (MCI/AD) population. Despite the lack of high positive predictive value (PPV) in asymptomatic individuals, *APOE* testing may still hold personal utility
Background on *APOE* alleles and cardiovascular risk	ε4: Dose-dependent risk factor for coronary artery disease and elevated LDL-cholesterol (Ison et al., 2025) ε2/ ε2: Increased risk of hyperlipoproteinemia type III (Ison et al., 2025)	Risk may be able to be modified through lifestyle changes
Background information on *APOE* inheritance	Individual with one *APOE* ε4: Each child has 50% risk to inherit an ε4^1^ Each sibling has 50% risk to inherit an ε4 Individual with two *APOE* ε4s: Each child has 100% risk to inherit an ε4^2^ Each sibling would have a 75% risk to have an ε4 (25% risk to inherit 2 ε4s, a 50% risk for 1 ε4) Each parent would be an obligate carrier	An ε4 will presumably have been inherited from a parent, a grandparent and up through the generations
Pre-test counseling	Review of medical and family history to assess risk and determine if additional genetic testing is indicated since there are multiple types of dementia that are associated with different genes Benefits, risks and limitations of testing Inquire about psychological status and determine if therapeutic support is needed Provision of supportive resources (e.g., Alzheimer’s Association)	Symptomatic: Implications of *APOE* genotype for both anti-amyloid considerations (e.g., ARIA risk) and familial AD risk Asymptomatic: Insurance implications and potential for genetic discrimination (including discussion of country-specific legislation and gaps in protection, e.g., GINA in the U. S.) Opportunities for mitigating risk by lifestyle changes Potential research involvement
Post-test counseling	Review background information and risk as indicated Review of results and provide patient with a copy of the report Implications of *APOE* genotype for individual and family members Patients should be encouraged to keep informed about research and clinical updates (e.g., Alzheimer’s Association) Review APOE risk for CVD and discuss lifestyle modifications	Symptomatic: If ε4 carrier, may impact eligibility for anti-amyloid therapy Asymptomatic: If develop any cognitive concerns, recommend evaluation by their medical provider, regardless of APOE status : Post-test counseling is recommended to help patients understand the implications of their *APOE* genotype

1 If the individual’s partner has one ε4, then the risks for each child are: 2 ε4s (25%), 1 ε4 (50%) and 0 ε4s (25%) If the individual’s partner has two ε4s, then the risks for each child are: 2 ε4s (50%), 1 ε4 (100%).

2 If the individual’s partner has one ε4, then the risks for each child are: 2 ε4s (50%), 1 ε4 (100%) If the individual’s partner has two ε4s, then the risks for each child are: 2 ε4s (100%).

For any individual considering *APOE* testing, the education and counseling content covered in this Perspective and in Table 1 are key to understanding its limitations and nuances and should be conveyed by the healthcare provider or through referral to a genetic counselor. After provision of this information, if a symptomatic patient or an asymptomatic patient with a first-degree affected relative and/or known *APOE* ε4 in the family subsequently wishes to proceed with *APOE* testing, then we advocate this should be a clinical option and incorporated into guidelines. It is preferable for individuals to have pre- and post-test counseling with Table 1 points covered rather than proceeding with direct-to-consumer (DTC) genetic testing without a clinician’s involvement. Furthermore, DTC testing raises the concern of a commercial entity having private patient healthcare information and the potential for data-sharing.

### Changing landscape: pros and cons of asymptomatic *APOE* testing

Though historically, *APOE* testing has not been recommended for asymptomatic individuals (American College of Medical Genetics/American Society of Human Genetics Working Group, 1995; Relkin et al., 1996; Waldemar et al., 2007; Goldman et al., 2011), we believe it is time to reconsider for individuals who have a first-degree affected relative and/or known *APOE* ε4 in the family. If a first-degree relative is affected, the risk for AD is increased to 20–25%, or higher if multiple family members have late-onset AD (Bird, 2008; Cannon-Albright et al., 2019). The increased AD risks due to having an *APOE* ε4 are noted above. While concern is understandable, an affected second-degree relative (e.g., grandparent) generally would not be an indication for *APOE* testing. A grandchild’s risk is not significantly increased (though risk figures are not readily available) and would be dependent on whether their parent develops AD.

While positive *APOE* ε4 carrier status currently has no impact on medical care for an asymptomatic individual, studies have shown that it can hold personal utility and there is high interest about learning AD risk (Marshe et al., 2019; Ryan et al., 2021). Individuals have made lifestyle changes (i.e., exercise, diet) and engaged in future planning after learning about their *APOE* ε4 carrier status (Chao et al., 2008; Largent et al., 2021). Knowledge of positive *APOE* ε4 carrier status may also encourage engagement in AD research studies or clinical trials focused on prevention (Iacono, 2025). Furthermore, though *APOE* is an AD risk factor and is not ready for utilization in population-level screening, the field is shifting to earlier prediction and potentially prevention of AD; *APOE* genotyping is one component that can be used to work toward achieving this goal.

Research mostly on individuals with a family history of AD has shown that *APOE* testing in asymptomatic individuals is relatively safe with few adverse psychological outcomes when accompanied by pre- and post-test counseling (Cassidy et al., 2008; Green et al., 2009; Largent et al., 2021; Alber et al., 2022; Langbaum et al., 2025). It has also been shown to be safe when performed by condensed counseling approaches (Roberts et al., 2012; Green et al., 2015) and by telephone counseling (Christensen et al., 2018). Furthermore, *APOE* disclosure for individuals with mild cognitive impairment (MCI) similarly did not show significant increases in anxiety and depression (Christensen et al., 2020) when accompanied by pre- and post-test counseling. In 2017, the FDA considered *APOE* testing safe enough to issue approval for the company 23andMe to offer this testing DTC (Maxmen, 2017).

Another reason to reconsider offering *APOE* testing is the association between *APOE* and cardiovascular risk (Table 1). Inclusion of *APOE* in routine CVD screening for dyslipidemia is recommended (Sturm et al., 2018) and the importance of counseling is noted if ε4 status is reported or revealed (Ison et al., 2025). We recommend clinicians counsel on both cardiovascular and AD implications when ordering *APOE* testing in any setting. Studies on learning about *APOE-*associated risk for both AD and CVD are limited but a randomized trial suggested that it is psychologically safe to disclose these risks with pre- and post-test counseling, and led to some study participants making health behavior changes when provided with risk modification information for CVD (Christensen et al., 2016). Additionally, research indicates that having an *APOE* ε4 may reduce response to statins and needs to be considered when prescribing statins (Asiimwe et al., 2026).

A potential concern for asymptomatic *APOE* disclosure is whether individuals can comprehend results or will misinterpret the significance of *APOE* being a risk factor and not a deterministic gene. A 2021 study demonstrated that cognitively unimpaired *APOE* ε4 carriers who received pre- and post- test counseling understood the implications of their results (Largent et al., 2021). However, *APOE* status may influence risk perception with Langbaum et al. (2025) showing that *APOE* ε4 homozygotes possessed a higher perceived AD risk from their pre-test baseline compared to *APOE* ε4 heterozygotes and non-carriers (Langbaum et al., 2025). In addition to risk perception, knowledge of one’s increased AD risk due to *APOE* ε4 may impact current cognitive functioning in asymptomatic individuals (Lineweaver et al., 2014) and self-perceptions and attitudes about one’s memory (Largent et al., 2021; Iacono, 2025).

Other commonly cited harms of *APOE* ε4 disclosure to asymptomatic individuals include the immediate psychological reactions and potential long-term psychological burden of having an increased risk of AD. These harms can be exacerbated by the method of result disclosure. Many people, including well-known celebrities such as actor Chris Hemsworth, have sought DTC *APOE* testing (Breznican, 2022; Hamilton, 2025). This generally has been done in the absence of any counseling and some individuals report adverse psychological reactions (depression, anxiety) when learning their results without clinician involvement (Messner, 2011; Koeller et al., 2017; Zallen, 2018; Steenhuysen, 2023; Schonbek, 2024). The timing of learning about *APOE* results is also a consideration and Iacono (2025) discusses pros and cons of disclosure in early adulthood. Ethical questions also exist about whether knowing one’s *APOE* status could lead to stigma and discrimination for certain types of insurance and in the workplace (Iacono, 2025).

### Pre- and post-test *APOE* counseling

Pre- and post-test counseling should include information on *APOE*’s association with AD and CVD risk, how *APOE* is inherited, and the personal and familial implications of knowing one’s *APOE* status (Table 1). Common clinical scenarios of *APOE* testing, topics to address (including suggested wording for risk communication, ascertaining risk perceptions) and recommended next steps are presented in a paper by Stites et al. (2022) and *APOE* counseling considerations, approaches and tips are presented in Zampatti et al. (2025).

We recommend pre- and post-test counseling for higher risk asymptomatic individuals interested in *APOE* testing. Counseling can be facilitated by genetic counselors, specialists, or primary care providers (Stites et al., 2022). Symptomatic individuals may also benefit from meeting with a genetic counselor if they want further discussion or need additional education for decision-making (Rumbaugh and Chan, 2025; Zampatti et al., 2025).

For all patients, ascertaining family history is important for determining risk, appropriate genetic testing, and familial implications. This includes obtaining age of onset/age at diagnosis, the presenting symptoms and any comorbidities to rule out other neurological/genetic conditions. For deceased individuals, it is important to determine if an autopsy was performed to confirm the type of dementia. While the focus for symptomatic individuals may be largely on the *APOE* gene for treatment decisions, a multigene panel of autosomal dominant dementia genes may be indicated if the patient has early-onset symptoms and/or a positive family history of early-onset AD/dementia.

### *APOE* genetic counseling for asymptomatic individuals

In addition to the above pre- and post-test counseling and Table 1 information, there are specific considerations for asymptomatic individuals. Family history should be reviewed to evaluate risk of AD versus a different dementia and to assess the mode of inheritance in the family (Goldman et al., 2011; Pagano et al., 2024). It is not unusual to have multiple affected relatives since up to 25% of all AD is familial of which 95% is late-onset (Bird, 2018). The family history assessment could significantly impact the genetic testing that is considered; the at-risk individual may ask incorrectly for *APOE* testing instead of testing for autosomal dominant dementia genes. Other risk factors should also be ascertained (e.g., alcohol, lack of sleep, heart disease) and patients should be encouraged to discuss lifestyle modifications with their primary healthcare provider to reduce AD risk.

Clinicians should stress that *APOE* ε4 is one of many genetic and environmental factors contributing to AD risk and that available risk figures are based primarily on individuals of European ancestry; further research is needed to clarify lifetime risk estimates for other ethnicities. Asymptomatic individuals should understand that AD is not inevitable if an *APOE* ε4 variant is identified. Just 60% of individuals with AD have an *APOE* ε4 variant (Van Cauwenberghe et al., 2016). Furthermore, if an affected parent’s ε4 is not inherited, AD risk is partially reduced but not eliminated.

Counseling should also explore the potential psychological impact of knowledge of one’s *APOE* status. Though most individuals find the information helpful and cope well with their results, as previously noted, some may have adverse psychological reactions, especially when pre-test genetic counseling was not received. Referral to a mental health professional may be advisable.

There are insurance considerations that need to be discussed during counseling asymptomatic individuals. Health insurance may not cover the genetic testing costs if asymptomatic. Other types of insurance can be impacted for asymptomatic individuals and genetic non-discrimination laws differ by country (Prince, 2019; Joly et al., 2020). In the United States, the Genetic Information Non-Discrimination Act (GINA) protects against discrimination in employment and for health insurance with some caveats but does not cover life, disability or long-term care insurance (Genetic Discrimination-National Human Genome Research Institute, 2025). Helpful information on GINA is available at https://ginahelp.org/

Finally, asymptomatic individuals should be aware that testing of other biomarkers in blood, cerebrospinal fluid, and brain amyloid PET scans provide stronger AD prognostic and diagnostic evidence (Bouwman et al., 2022; Chapleau et al., 2022; Mielke and Fowler, 2024). In fact, usage of biomarker-based testing is expanding and the FDA recently approved the first blood-based AD biomarker test (Rubin, 2025). However, caution needs to be given to the potential for over-diagnosis solely based on a single biomarker or genetic test due to limitations of test sensitivity and specificity (Alzforum, 2025). It is important in developing guidance to look beyond just *APOE* testing to the future usage of these biomarker-based tests in asymptomatic individuals interested in ascertaining AD risk.

### Challenges of implementing clinical *APOE* testing for asymptomatic individuals

There are practice implications for expanding access to clinical *APOE* testing given the potential high interest. Clinicians in different clinical specialties and work settings can use the information in this Perspective to inform patients, facilitate decision-making, and streamline the communication of information. While neurologists, cardiologists, or primary care providers can generally convey the risk information discussed in this Perspective and order testing, genetic counselors involvement is recommended in certain clinical situations requiring extended discussion (e.g., autosomal dominant family history), especially with asymptomatic patients (Ritchie et al., 2024). Given patient volumes, the use of digital tools (e.g., chatbots) to assist with *APOE* education may be helpful and allow clinicians time to focus on risk assessment, decision-making, and interpretation of test results (Shickh et al., 2021; Erickson et al., 2025; Wood et al., 2025).

## Conclusion

*APOE* testing is an important component of risk assessment for AD and can yield results that are useful for both symptomatic and asymptomatic individuals. For symptomatic individuals, results can impact clinical care and provide risk information for family members. For asymptomatic individuals, there is the opportunity to clarify one’s AD risk, information which can be used to potentially make lifestyle modifications and inform future planning. Therefore, while *APOE* testing is neither diagnostic nor predictive for AD, it can hold value in patient care and personal utility for decision-making and life planning.

As experienced neurogenetic counselors, we frequently receive requests for *APOE* testing from asymptomatic patients and believe individuals with a first-degree affected relative and/or known *APOE* ε4 in the family should have the option of clinical testing with genetic counseling rather than DTC testing without it. However, wider access should not be implemented until guidance is provided by the neurology and genetics communities. The AD field is expanding beyond *APOE* to the use of other biomarkers. An update in guidance is needed given the increasing use of *APOE* testing in treatment decisions and would be particularly timely since AD polygenic risk scores (PRS) and testing for other AD biomarkers are anticipated to be introduced for asymptomatic individuals (Baker and Escott-Price, 2020; Grill, 2025). With multiple landscape changes and the shift toward earlier identification of individuals at-risk for AD, we think it is time as a medical community to discuss expanding clinical *APOE* genetic testing access and update guidelines.

## Data Availability

The original contributions presented in the study are included in the article. Further inquiries can be directed to the corresponding author.
